# CPR knowledge among rural grassroots healthcare workers in Xinjiang, China: a cross-sectional analysis

**DOI:** 10.3389/fmed.2025.1697798

**Published:** 2025-11-21

**Authors:** Jin Ma, Liuniu Kuai, Xiaolong Zhu, Qi Tang, Shifang Liu, Weiwei Zhou

**Affiliations:** 1 Department of Emergency Medicine, The People’s Hospital of Atushi City, Atushi, China; 2 Department of Urology Surgery, The People’s Hospital of Atushi City, Atushi, China; 3 Department of Cardiovascular Medicine, The People’s Hospital of Atushi City, Atushi, China

**Keywords:** out-of-hospital cardiac arrest, external defibrillator, township household health education instructors, cardiopulmonary resuscitation, cross-sectional analysis

## Abstract

Out-of-hospital cardiac arrest (OHCA) survival depends on rapid recognition, high-quality chest compressions, and early use of an automated external defibrillator (AED). Township household health education instructors in rural China are grassroots healthcare workers and are often the first reachable trained personnel during emergencies; however, their readiness to perform cardiopulmonary resuscitation (CPR) is undercharacterized. We conducted a cross-sectional analysis of a programmatic survey involving 235 instructors in Xinjiang, China. The survey instrument captured demographics, training exposure and its recency, AED awareness, item-level CPR knowledge, rescue willingness, and preferences. We derived a composite readiness score (0–100) and a strict overall accuracy score by averaging seven item-level correctness indicators. Group differences were assessed using chi-squared, Fisher’s exact, or Kruskal–Wallis tests, with false discovery rate (FDR)-adjusted *post hoc* comparisons. Readiness correlates were examined using ordinary least squares (OLS) regression. The participants were predominantly women (97.4%) with a mean age of 37.4 ± 7.1 years; 86.4% were Uyghur, 12.3% Kyrgyz, and 1.3% Han. In total, 87.7% of the participants received CPR training, and 80.4% reported AED awareness. The mean readiness score was 79.4 ± 19.0, and the strict overall accuracy was 62.2 ± 25.6%. Item-level correct rates were as follows: “golden time,” 77.4%; compression location, 73.2%; compression rate, 63.4%; indication, 62.1%; compression depth, 56.6%; AED timing, 56.2%; and all respiration steps, 46.8%. The trained participants showed higher readiness (83.7 ± 14.4 vs. 48.4 ± 18.9; *p* < 0.0001) and accuracy (0.65 vs. 0.42; *p* < 0.0001) compared to the untrained participants. Readiness varied by ethnicity (*p* = 0.0096; Uyghur > Kyrgyz, FDR = 0.050) and education (*p* = 0.00064). In the OLS model, having any prior training (+23.9; *p* < 0.0001), self-rated “very clear” knowledge (+13.7; *p* < 0.0001), and AED awareness (+5.0; *p* = 0.019) independently increased readiness, whereas middle-school education (−9.10; *p* = 0.0095) and married status (−7.77; *p* = 0.010) decreased readiness. The findings indicate generally favorable readiness with identifiable gaps, supporting low-dose, high-frequency, bilingual, hands-on refresher training tailored to rural Xinjiang.

## Introduction

Out-of-hospital cardiac arrest (OHCA) represents a universally time-critical emergency where survival depends on rapid recognition, high-quality cardiopulmonary resuscitation (CPR), and early defibrillation (1–8). Despite advances in resuscitation science, substantial rural–urban disparities in OHCA survival persist worldwide (9–13). These disparities reflect not only differences in emergency medical service (EMS) response times—which can exceed 30 min in remote areas compared to 8–10 min in urban centers—but also gaps in bystander CPR rates and public-access defibrillator availability. Evidence from population studies and meta-analyses across multiple continents demonstrates that bystander CPR and timely automated external defibrillator (AED) use substantially improve neurological outcomes and survival. However, the implementation of these evidence-based interventions remains inconsistent in resource-limited and geographically isolated settings (14–26).

In rural and remote settings around the world, formal EMS systems face significant geographic and temporal barriers. When ambulance transport times exceed the critical 4–8-min window for effective resuscitation, community health workers (CHWs) become de facto first responders. These frontline personnel—known variously as Accredited Social Health Activists (ASHAs) in India (27–29), Health Extension Workers in Ethiopia (30, 31), Community Health Agents in Brazil (32), and village doctors in China—provide primary health education, chronic disease management, and emergency response in underserved populations. Current resuscitation guidelines emphasize comprehensive, customized CPR training; however, empirical data characterizing the preparedness of CHWs to recognize and manage cardiac arrest remain remarkably sparse, particularly in multiethnic and multilingual regions where cultural and linguistic factors may compound implementation challenges (33–35).

China’s tiered rural health system offers a valuable perspective for examining these global challenges. Township household health education instructors—community-based health educators who conduct home visits for chronic disease management and health promotion, functionally equivalent to CHWs in other contexts—represent the most accessible trained personnel during medical emergencies in villages that are often located hours away from the nearest hospital.

The Xinjiang Uyghur Autonomous Region in northwestern China exemplifies the severity of these challenges. Spanning 1.66 million km^2^, Xinjiang is characterized by vast distances, sparse population density (15 persons/km^2^ versus the national average of 145/km^2^), and profound ethnic and linguistic diversity. The population comprises Uyghur (45%), Han Chinese (42%), and Kyrgyz, along with other minority groups (13%). As a result, health education materials are needed in Uyghur, Kyrgyz, and Mandarin Chinese. In Atushi, a prefecture in southern Xinjiang bordering Kyrgyzstan, some villages are located 3–5 h by road from the nearest county hospital, and township health centers may employ only approximately 30 clinicians to serve areas with 10,000 residents (36, 37). Emergency response times routinely exceed 30 min, making township health education instructors the most readily available trained personnel during the critical first minutes following a cardiac arrest.

Therefore, we conducted this cross-sectional analysis with three objectives: (1) to characterize baseline CPR knowledge and readiness among township household health education instructors in rural Xinjiang; (2) to identify demographic and training-related factors associated with knowledge gaps; and (3) to inform the development of targeted, culturally appropriate CPR training programs for this workforce. In addition to characterizing this specific workforce, our findings may guide CPR training strategies for community health workers in other geographic and cultural contexts where similar barriers to emergency preparedness exist.

## Materials and methods

### Study design and setting

We conducted a cross-sectional analysis of a programmatic survey administered to township household health education instructors in Atushi, Xinjiang, China. These instructors are employed by township health centers or community health service centers and supervised by the public health department. Their primary responsibilities mirror those of CHWs globally: conducting household visits to provide health education on chronic disease management (hypertension, diabetes), infectious disease prevention, and emergency preparedness. Community members access services through instructor-initiated household visits or by seeking care at health centers. In medical emergencies, instructors are often contacted by community members before or concurrently with calls to formal emergency services, making them de facto first responders.

### Participants and data collection

Eligible participants included all active township household health education instructors working in Atushi. The sample size was determined using a complete enumeration approach: all 235 active instructors in the region at the time of the survey were invited, and all consented, providing a census that eliminates sampling error within the defined population and ensures adequate power to detect effect sizes in group comparisons. The survey instrument was developed in Mandarin Chinese—the language of health education delivery in the region—although participants’ primary languages varied (Uyghur, Kyrgyz, or Chinese). Response options were aligned with international CPR guideline content, and responses were subsequently mapped to English labels for analysis and reporting.

The instrument collected information on demographics (gender, age, ethnicity, marital status, and education), training exposure indicators (any CPR training and recency), AED awareness, experience witnessing cardiac arrest, worry about emergencies, rescue willingness, perceived training necessity, and preferences for training formats and methods.

### Measures and operational definitions

The survey instrument was developed using a multistage process based on the 2020 American Heart Association CPR guidelines and adapted for the Chinese context in consultation with emergency medicine specialists and public health educators. The Chinese-language instrument was tested through cognitive interviews with five township health workers to assess comprehension and cultural appropriateness, with minor wording adjustments made based on feedback. Although formal psychometric validation was not conducted due to resource constraints, content validity was ensured through expert review and alignment with international guidelines.

CPR knowledge items mirrored core adult basic life support concepts, including indications for initiating CPR, the “golden time” for intervention, chest-compression location, chest-compression depth, chest-compression rate, timing of AED use, and a multi-select list of artificial respiration steps. Single-choice items were scored as correct if they matched guideline-concordant responses. The respiration sequence was scored in two ways: a strict “all-correct” indicator and a partial score ranging from 0 to 1, proportional to the number of correct steps selected.

Training exposure was dichotomized as “Trained” if the respondents reported either formal training (e.g., hospital or Red Cross) or self-study via videos/books; otherwise, they were classified as “Untrained.” Training recency was categorized as “≤6 months,” “6–12 months,” “1–2 years,” and “>2 years,” with “No training” assigned if the respondent reported no training or did not report a training time.

We derived two summary outcomes. The strict overall knowledge accuracy score was the mean of seven item-level correctness indicators, covering indications, golden time, compression location, compression depth, compression rate, AED timing, and respiration steps. The composite readiness score (0–100) integrated self-rated CPR knowledge (maximum 30 points), training exposure (maximum 20 points), correctness on four key knowledge items (maximum 30 points), and rescue willingness (maximum 20 points).

### Statistical analysis

Categorical variables were summarized using counts and percentages, while continuous variables were described with means and standard deviations and with medians and interquartile ranges as supplementary descriptors. Baseline comparisons between the trained and untrained participants were performed using chi-squared tests for categorical variables, Fisher’s exact test for sparse 2 × 2 tables, and the Kruskal–Wallis test for continuous variables. To assess group differences in readiness and strict overall accuracy for single-choice variables, the Kruskal–Wallis test was used and, when significant, the pairwise Mann–Whitney U test with Benjamini–Hochberg false discovery rate (FDR) adjustment was conducted. Item-level accuracy across subgroups (training status, gender, ethnicity, age group, education, and marital status) was compared using chi-squared or Fisher’s exact tests (Supplementary Table 1).

To identify independent correlates of readiness, an ordinary least squares (OLS) regression with HC3 robust standard errors was used. Predictors included training status, age, gender, ethnicity, marital status, education, AED awareness, worry about emergencies, and self-rated CPR knowledge. Statistical significance was defined as a two-sided *p*-value of < 0.05. Analyses were conducted using Python 3.9.

## Results

### Participant characteristics

We analyzed 235 township household health education instructors (Table 1). The cohort was predominantly female (*N* = 229, 97.4%), with a mean age of 37.4 ± 7.1 years. The majority of the participants identified as Uyghur (*N* = 203, 86.4%), with smaller proportions identifying as Kyrgyz (*N* = 29, 12.3%) and Han Chinese (*N* = 3, 1.3%). The majority of the participants were married (*N* = 211, 89.8%), and educational attainment was primarily junior college (*N* = 110, 46.8%) or high/technical school (*N* = 75, 31.9%), with 13.6% holding a bachelor’s degree and 7.7% completing middle-school education. These characteristics reflect the staffing composition of village-level public health personnel in rural Xinjiang.

**Table 1:** Demographic information of the participants and the characteristics of the questionnaire (*N* = 235).

Characteristics	Overall	Untrained	Trained	Test	*P*-value
Gender				Fisher’s exact test	0.999
Women	229 (97.4%)	29 (100.0%)	200 (97.1%)		
Men	6 (2.6%)	0 (0.0%)	6 (2.9%)		
Age group				Chi-squared test	0.6844
18–29	26 (11.1%)	3 (10.3%)	23 (11.2%)		
30–39	124 (52.8%)	18 (62.1%)	106 (51.5%)		
40–49	70 (29.8%)	6 (20.7%)	64 (31.1%)		
50+	15 (6.4%)	2 (6.9%)	13 (6.3%)		
Ethnicity				Chi-squared test	0.01347
Han	3 (1.3%)	1 (3.4%)	2 (1.0%)		
Kyrgyz	29 (12.3%)	8 (27.6%)	21 (10.2%)		
Uyghur	203 (86.4%)	20 (69.0%)	183 (88.8%)		
Marital status				Chi-squared test	0.4947
Divorced	7 (3.0%)	2 (6.9%)	5 (2.4%)		
Married	211 (89.8%)	26 (89.7%)	185 (89.8%)		
Single	16 (6.8%)	1 (3.4%)	15 (7.3%)		
Widowed	1 (0.4%)	0 (0.0%)	1 (0.5%)		
Education				Chi-squared test	0.00065
Bachelor’s degree	32 (13.6%)	11 (37.9%)	21 (10.2%)		
High/technical school	75 (31.9%)	5 (17.2%)	70 (34.0%)		
Junior college	110 (46.8%)	11 (37.9%)	99 (48.1%)		
Middle school	18 (7.7%)	2 (6.9%)	16 (7.8%)		
CPR knowledge				Chi-squared test	0.000679
General	56 (23.8%)	12 (41.4%)	44 (21.4%)		
No knowledge	8 (3.4%)	3 (10.3%)	5 (2.4%)		
Only heard	21 (8.9%)	5 (17.2%)	16 (7.8%)		
Very clear	150 (63.8%)	9 (31.0%)	141 (68.4%)		
Know what AED is				Chi-squared test	8.07E-13
No	46 (19.6%)	20 (69.0%)	26 (12.6%)		
Yes	189 (80.4%)	9 (31.0%)	180 (87.4%)		
Golden time				Chi-squared test	0.001639
Not sure	5 (2.1%)	2 (6.9%)	3 (1.5%)		
Over 10 min	26 (11.1%)	8 (27.6%)	18 (8.7%)		
Within 4 min	182 (77.4%)	15 (51.7%)	167 (81.1%)		
Within 8 min	22 (9.4%)	4 (13.8%)	18 (8.7%)		
Compression location				Chi-squared test	3.46E-05
Chest center	172 (73.2%)	13 (44.8%)	159 (77.2%)		
Left chest	44 (18.7%)	11 (37.9%)	33 (16.0%)		
Not sure	7 (3.0%)	4 (13.8%)	3 (1.5%)		
Right chest	12 (5.1%)	1 (3.4%)	11 (5.3%)		
Compression depth				Chi-squared test	0.000114
3–4 cm	80 (34.0%)	11 (37.9%)	69 (33.5%)		
5–6 cm	133 (56.6%)	11 (37.9%)	122 (59.2%)		
>6 cm	14 (6.0%)	2 (6.9%)	12 (5.8%)		
Not sure	8 (3.4%)	5 (17.2%)	3 (1.5%)		
Compression rate				Chi-squared test	0.000768
100-120/min	149 (63.4%)	16 (55.2%)	133 (64.6%)		
60-100/min	72 (30.6%)	8 (27.6%)	64 (31.1%)		
>120/min	8 (3.4%)	1 (3.4%)	7 (3.4%)		
Not sure	6 (2.6%)	4 (13.8%)	2 (1.0%)		
When to use AED				Chi-squared test	1.58E-05
Immediate analysis and shock	132 (56.2%)	12 (41.4%)	120 (58.3%)		
Not sure	13 (5.5%)	7 (24.1%)	6 (2.9%)		
Use after 2 min of CPR	90 (38.3%)	10 (34.5%)	80 (38.8%)		
Witnessed SCA (public/home)				Chi-squared test	0.04477
No	113 (48.1%)	19 (65.5%)	94 (45.6%)		
Yes	122 (51.9%)	10 (34.5%)	112 (54.4%)		
Worry about emergencies				Chi-squared test	0.044587
Never	15 (6.4%)	5 (17.2%)	10 (4.9%)		
Often	119 (50.6%)	10 (34.5%)	109 (52.9%)		
Seldom	31 (13.2%)	4 (13.8%)	27 (13.1%)		
Sometimes	70 (29.8%)	10 (34.5%)	60 (29.1%)		
Rescue willingness				Chi-squared test	3.67E-05
Need legal protection	20 (8.5%)	5 (17.2%)	15 (7.3%)		
Not willing	4 (1.7%)	3 (10.3%)	1 (0.5%)		
Willing and competent	171 (72.8%)	13 (44.8%)	158 (76.7%)		
Willing but worried	40 (17.0%)	8 (27.6%)	32 (15.5%)		
Skill mastery after training				Chi-squared test	1.11E-49
Almost forgotten	4 (1.7%)	0 (0.0%)	4 (1.9%)		
Competent independently	104 (44.3%)	0 (0.0%)	104 (50.5%)		
Know theory only	20 (8.5%)	0 (0.0%)	20 (9.7%)		
No training	29 (12.3%)	29 (100.0%)	0 (0.0%)	Chi-squared test	
Remember steps but rusty	78 (33.2%)	0 (0.0%)	78 (37.9%)	Chi-squared test	
Last training time (display rule)				Chi-squared test	1.11E-49
1–2 years	21 (8.9%)	0 (0.0%)	21 (10.2%)		
6–12 months	62 (26.4%)	0 (0.0%)	62 (30.1%)		
>2 years	11 (4.7%)	0 (0.0%)	11 (5.3%)		
No training	29 (12.3%)	29 (100.0%)	0 (0.0%)		
≤6 months	112 (47.7%)	0 (0.0%)	112 (54.4%)		
Volunteer willingness				Chi-squared test	2.42E-05
Depends on time	30 (12.8%)	8 (27.6%)	22 (10.7%)		
Not now	7 (3.0%)	4 (13.8%)	3 (1.5%)		
Willing	198 (84.3%)	17 (58.6%)	181 (87.9%)		
Training necessity				Chi-squared test	0.02275
Average	15 (6.4%)	5 (17.2%)	10 (4.9%)		
Necessary	44 (18.7%)	9 (31.0%)	35 (17.0%)		
Not necessary	1 (0.4%)	0 (0.0%)	1 (0.5%)		
Not very necessary	2 (0.9%)	0 (0.0%)	2 (1.0%)		
Very necessary	173 (73.6%)	15 (51.7%)	158 (76.7%)		
Age (years)	37.4 ± 7.1	36.9 ± 7.0	37.4 ± 7.1	Kruskal–Wallis test	0.3961
Readiness score	79.4 ± 19.0	48.4 ± 18.9	83.7 ± 14.4	Kruskal–Wallis test	1.8E-14
Overall accuracy (strict)	0.6 ± 0.3	0.4 ± 0.3	0.7 ± 0.2	Kruskal–Wallis test	6.57E-06
Conscious with chest pain	13 (5.5%)	1 (3.4%)	12 (5.8%)	Chi-squared test	0.058559
Not sure	13 (5.5%)	4 (13.8%)	9 (4.4%)		
Pulse present but experiencing dyspnea	63 (26.8%)	11 (37.9%)	52 (25.2%)		
Unresponsive and not breathing normally	146 (62.1%)	13 (44.8%)	133 (64.6%)		

Subgroups with and without CPR training were compared.

The proportion of CPR training was high: 87.7% reported any CPR training and 75.3% reported formal training (Table 1). Training recency was favorable, with 47.7% trained within the past 6 months, 26.4% within 6–12 months, 8.9% within 1–2 years, and 4.7% more than 2 years prior; 12.3% reported no training. AED awareness was reported by 80.4% of the respondents. The majority of the participants (*N* = 122, 51.9%) had witnessed a cardiac arrest in a public setting or at home. Rescue willingness was high, with 72.8% indicating they were willing and felt competent to act, 17.0% willing but worried, 8.5% willing only with legal protection, and 1.7% not willing. The majority of respondents (*N* = 173, 73.6%) considered CPR training “very necessary,” with 18.7% rating it “necessary.”

### Overall readiness and knowledge performance

**Figure 1:**
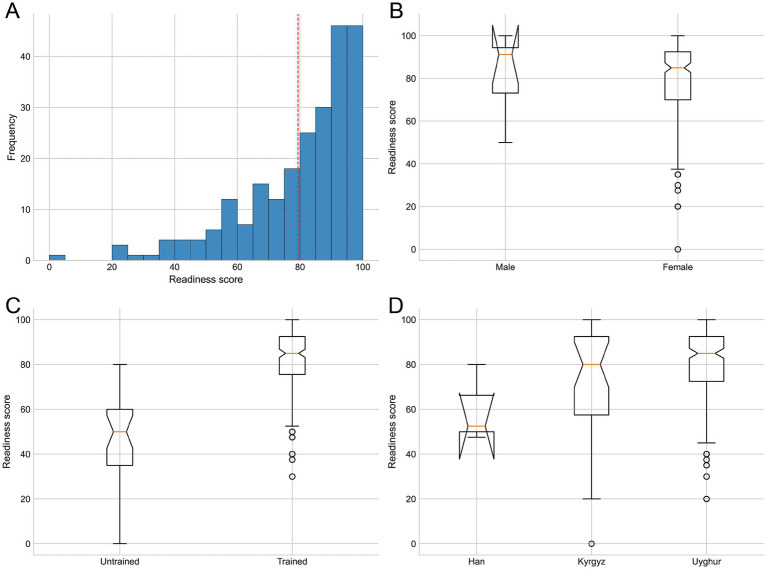
Statistical analysis of CPR readiness scores. **(A)** Overall distribution (histogram). **(B)** Group comparisons by gender. **(C)** Group comparisons by training status. **(D)** Group comparisons by ethnicity.

The composite readiness score averaged 79.4 ± 19.0, with a median of 85.0 and an interquartile range of 70.0 to 92.5 (Table 1, Figure 1A). The strict overall knowledge accuracy score averaged 62.2 ± 25.6%, with a median of 70% and an interquartile range of 40%–90% (Table 2). Correct identification of the “golden time” (77.4%) and adult compression location (73.2%) was relatively strong. Correct selection of the compression rate was 63.4% and of the indication to initiate CPR was 62.1%. Lower correct rates were observed for compression depth (56.6%) and AED timing (56.2%). The strict all-correct score for artificial-respiration steps was 46.8%.

**Table 2:** Accuracy of the questions in the questionnaire.

Item	Correct rate (%)
When CPR is needed	146 (62.1%)
Golden time	182 (77.4%)
Compression location	172 (73.2%)
Compression depth	133 (56.6%)
Compression rate	149 (63.4%)
When to use AED	132 (56.2%)
All respiration steps	110 (46.8%)

### Comparisons by training status

The trained participants outperformed the untrained peers across primary outcomes (Table 1). The readiness score was 83.7 ± 14.4 among the trained respondents versus 48.4 ± 18.9 among the untrained respondents, representing a highly significant difference by the Kruskal–Wallis test (*p* < 0.0001). The strict overall knowledge accuracy score was correspondingly higher among the trained respondents than among the untrained respondents (0.65 vs. 0.42; *p* < 0.0001). At the item level, training status was associated with higher correct rates for several knowledge items. Correct recognition of the “golden time” was 81.1% among the trained participants compared to 51.7% among the untrained participants (chi-squared, *p* = 0.00040). Correct identification of the compression location was 77.2% versus 44.8% (*p* = 0.00023). Correct indication for initiating CPR was 64.6% versus 44.8% (*p* = 0.040), compression depth 59.2% versus 37.9% (*p* = 0.030), and all-correct respiration steps 51.0% versus 17.2% (*p* = 0.00065). Differences between the trained and untrained participants were smaller for the compression rate (64.6% vs. 55.2%; *p* = 0.326) and AED timing (58.3% vs. 41.4%; *p* = 0.086). Training recency showed a graded association: readiness was highest among those trained within 6 months (87.2 ± 12.5) and lowest in the no-training group (48.4 ± 18.9), with an overall *p*-value of < 0.0001 (Kruskal–Wallis). Strict overall accuracy also differed by training recency (*p* < 0.0001).

### Group differences across demographic subgroups

Readiness varied by ethnicity (Kruskal–Wallis, *p* = 0.0096), with pairwise Mann–Whitney contrasts demonstrating higher readiness among the Uyghur respondents than the Kyrgyz respondents (mean 81.1 vs. 69.0; raw *p* = 0.0167; false discovery rate-adjusted *p* = 0.050) (Tables 3, 4, Figure 1). Although the Han respondents had a small sample size (*n* = 3) and hence imprecise estimates, their mean readiness was 60.0 ± 17.5. Strict overall accuracy also differed by ethnicity (*p* = 0.03996); pairwise comparisons showed that the Uyghur respondents outperformed the Kyrgyz respondents (FDR-adjusted *p* = 0.036). Education was associated with readiness (*p* = 0.0006368) and strict overall accuracy (*p* = 0.01698), with lower scores observed for the participants with a middle-school education compared to the junior college and high/technical groups. In contrast, neither gender nor age group was associated with readiness or strict overall accuracy.

**Table 3:** Differences in the readiness score across demographic subgroups.

Characteristics	*N*	Mean ± SD	Median [IQR]	Statistic	P-value
Gender				0.344615	0.557177
Women	229	79.3 ± 19.0	85.0 [70.0, 92.5]		
Men	6	82.5 ± 19.5	91.2 [73.1, 94.4]		
Age group				2.401081	0.493433
18–29	26	77.1 ± 20.2	80.0 [68.1, 91.9]		
30–39	124	81.2 ± 17.8	85.0 [72.5, 92.5]		
40–49	70	78.4 ± 18.8	82.5 [67.5, 92.5]		
50+	15	73.0 ± 26.2	75.0 [65.0, 92.5]		
Ethnicity				9.299792	0.009563
Uyghur	203	81.1 ± 17.2	85.0 [72.5, 92.5]		
Kyrgyz	29	69.0 ± 26.0	80.0 [57.5, 92.5]		
Han	3	60.0 ± 17.5	52.5 [50.0, 66.2]		
Marital status				3.721935	0.293098
Married	211	79.0 ± 19.0	85.0 [67.5, 92.5]		
Single	16	83.8 ± 19.8	86.2 [77.5, 100.0]		
Divorced	7	76.4 ± 18.0	80.0 [65.0, 87.5]		
Widowed	1	100.0	100.0 [100.0, 100.0]		
Education				17.22005	0.000637
Junior college	110	81.2 ± 19.6	85.0 [72.5, 95.0]		
High/technical school	75	83.3 ± 13.8	85.0 [77.5, 92.5]		
Bachelor’s degree	32	67.7 ± 22.3	67.5 [50.0, 86.9]		
Middle school	18	71.9 ± 18.5	76.2 [65.0, 84.4]		
Any CPR training				58.74336	1.8E-14
Trained	206	83.7 ± 14.4	85.0 [75.6, 92.5]		
Untrained	29	48.4 ± 18.9	50.0 [35.0, 60.0]		

**Table 4:** Differences in overall accuracy across demographic subgroups.

Characteristics	*N*	Mean ± SD	Median [IQR]	Statistic	*P*-value
Gender				0.080891	0.776093
Women	229	0.6 ± 0.3	0.7 [0.4, 0.9]		
Men	6	0.6 ± 0.3	0.6 [0.5, 0.8]		
Age group				2.485952	0.477835
30–39	124	0.6 ± 0.2	0.7 [0.5, 0.9]		
40–49	70	0.6 ± 0.3	0.6 [0.4, 0.9]		
18–29	26	0.6 ± 0.3	0.7 [0.4, 0.9]		
50+	15	0.6 ± 0.3	0.6 [0.5, 0.8]		
Ethnicity				6.439829	0.039958
Uyghur	203	0.6 ± 0.2	0.7 [0.5, 0.9]		
Kyrgyz	29	0.5 ± 0.3	0.6 [0.1, 0.7]		
Han	3	0.6 ± 0.4	0.4 [0.4, 0.7]		
Marital status				11.16125	0.010885
Married	211	0.6 ± 0.3	0.7 [0.4, 0.9]		
Single	16	0.8 ± 0.3	0.9 [0.7, 0.9]		
Divorced	7	0.7 ± 0.2	0.7 [0.6, 0.9]		
Widowed	1	1.0	1.0 [1.0, 1.0]		
Education				10.19511	0.016978
Junior college	110	0.6 ± 0.3	0.7 [0.4, 0.9]		
High/technical school	75	0.7 ± 0.2	0.7 [0.6, 0.9]		
Bachelor’s degree	32	0.6 ± 0.3	0.7 [0.4, 0.9]		
Middle school	18	0.4 ± 0.3	0.4 [0.3, 0.6]		
CPR knowledge				25.60384	1.15E-05
Very clear	150	0.7 ± 0.2	0.7 [0.6, 0.9]		
General	56	0.6 ± 0.3	0.6 [0.4, 0.9]		
Only heard	21	0.4 ± 0.2	0.4 [0.3, 0.6]		
No knowledge	8	0.4 ± 0.3	0.4 [0.1, 0.6]		
Know what AED is				17.87354	2.36E-05
Yes	189	0.7 ± 0.2	0.7 [0.6, 0.9]		
No	46	0.5 ± 0.3	0.5 [0.3, 0.7]		
Witnessed SCA (public/home)				0.428865	0.512547
Yes	122	0.6 ± 0.3	0.7 [0.4, 0.9]		
No	113	0.6 ± 0.3	0.7 [0.4, 0.9]		
Worry about emergencies				0.87944	0.830386
Often	119	0.6 ± 0.2	0.7 [0.4, 0.9]		
Sometimes	70	0.6 ± 0.3	0.6 [0.4, 0.9]		
Seldom	31	0.6 ± 0.2	0.7 [0.4, 0.9]		
Never	15	0.6 ± 0.4	0.7 [0.4, 0.9]		
Rescue willingness				12.35519	0.00626
Willing and competent	171	0.7 ± 0.2	0.7 [0.6, 0.9]		
Willing but worried	40	0.6 ± 0.3	0.6 [0.4, 0.9]		
Need legal protection	20	0.4 ± 0.3	0.4 [0.1, 0.6]		
Not willing	4	0.4 ± 0.5	0.3 [0.0, 0.7]		
Skill mastery after training				33.49004	9.48E-07
Competent independently	104	0.7 ± 0.2	0.7 [0.6, 0.9]		
Remember steps but rusty	78	0.6 ± 0.3	0.7 [0.6, 0.9]		
No training	29	0.4 ± 0.3	0.4 [0.3, 0.6]		
Know theory only	20	0.5 ± 0.3	0.5 [0.3, 0.8]		
Almost forgotten	4	0.3 ± 0.3	0.2 [0.1, 0.4]		
Last training time (display rule)				33.88413	7.87E-07
≤6 months	112	0.7 ± 0.2	0.7 [0.6, 0.9]		
6–12 months	62	0.6 ± 0.3	0.6 [0.4, 0.7]		
No training	29	0.4 ± 0.3	0.4 [0.3, 0.6]		
1–2 years	21	0.6 ± 0.3	0.7 [0.4, 0.9]		
>2 years	11	0.7 ± 0.2	0.7 [0.6, 0.8]		
Volunteer willingness				8.806914	0.012235
Willing	198	0.6 ± 0.2	0.7 [0.6, 0.9]		
Depends on time	30	0.6 ± 0.3	0.6 [0.3, 0.7]		
Not now	7	0.3 ± 0.3	0.4 [0.1, 0.5]		
Training necessity				16.42818	0.002495
Very necessary	173	0.7 ± 0.2	0.7 [0.6, 0.9]		
Necessary	44	0.6 ± 0.3	0.6 [0.4, 0.7]		
Average	15	0.4 ± 0.3	0.6 [0.3, 0.6]		
Not very necessary	2	0.3 ± 0.2	0.3 [0.2, 0.4]		
Not necessary	1	0.1 ± nan	0.1 [0.1, 0.1]		
Any CPR training				20.31399	6.57E-06
Trained	206	0.7 ± 0.2	0.7 [0.6, 0.9]		
Untrained	29	0.4 ± 0.3	0.4 [0.3, 0.6]		

### Multivariable correlates of readiness

In OLS regression, independent correlates of readiness included training, self-rated knowledge, AED awareness, education, and marital status (Table 5). Any CPR training was associated with a 23.88-point increase in readiness (*p* < 0.0001) after adjusting for covariates. Self-rated “very clear” CPR knowledge was associated with a 13.72-point increase (*p* < 0.0001), whereas “only heard” and “no knowledge” categories were associated with 15.86- and 25.90-point decreases, respectively (both *p* < 0.0001), relative to reference categories. AED awareness (Yes) was associated with a 4.98-point increase in readiness (*p* = 0.019). Middle-school education was associated with a 9.10-point decrease relative to a bachelor’s degree (*p* = 0.0095). Married status was associated with a 7.77-point decrease relative to other categories (*p* = 0.010). Gender, age, and ethnicity were not significant after adjustment, indicating that the primary drivers of readiness were training exposure and knowledge-related factors, with education and marital status identifying subgroups potentially in need of additional support.

**Table 5:** Independent predictors associated with CPR readiness based on OLS regression.

Variable	Coef	SE	*t*	*P*-value
Intercept	43.464	10.470	4.151	3.31E-05
Gender				
Men	1.459	4.214	0.346	0.729276
Ethnicity				
Kyrgyz	9.677	8.537	1.133	0.257029
Uyghur	8.747	8.339	1.049	0.29423
Marital				
Married	−7.770	3.023	−2.571	0.010153
Single	−6.278	4.147	−1.514	0.13004
Widowed	1.139	64.085	0.018	0.985815
Education				
High/technical school	−1.116	2.518	−0.443	0.657802
Junior college	−1.262	2.584	−0.488	0.625366
Middle school	−9.098	3.507	−2.594	0.009489
AED awareness				
Yes	4.984	2.128	2.342	0.019176
Worry				
Often	3.732	3.454	1.080	0.279961
Seldom	4.029	3.765	1.070	0.284559
Sometimes	2.823	3.641	0.775	0.438166
CPR knowledge				
No knowledge	−25.905	5.433	−4.768	1.86E-06
Only heard	−15.856	3.504	−4.524	6.06E-06
Very clear	13.722	1.802	7.613	2.68E-14
Trained	23.880	2.757	8.663	4.59E-18
Age	0.040	0.119	0.335	0.737422

### Training preferences and perceived helpful methods

Training preferences aligned with contemporary resuscitation education approaches. The respondents favored regular in-person practical sessions (71.9%), expert lectures with certification (70.6%), and online courses with simulated practice (68.9%), with additional interest in community self-training equipment (55.3%) and emergency knowledge competitions (48.9%) (Table 6). Perceived helpful training methods included theoretical instruction (79.1%), hands-on practice (76.2%), model demonstrations (60.0%), VR simulation (39.1%), video case studies (52.8%), and small-group discussions (42.6%).

**Table 6:** Training preferences and perceived helpful methods.

Variable (multi)	Count	Percent
Factors affecting CPR success (multi-select)		
Standardization of compression depth and frequency	173	73.6
Rescue time	169	71.9
Patient’s age or underlying diseases	114	48.5
Is there an AED on site	114	48.5
Preferred training methods (multi-select)		
Theoretical explanation	186	79.1
Model demonstration	141	60
Practical exercise	179	76.2
VR simulation	92	39.1
Video case	124	52.8
Group discussion	100	42.6
Artificial respiration steps (multi-select)		
Open the airway (head-up and chin-lifting method)	195	83
Breathe for one second each time and observe the rise and fall of the chest cavity	0	0
Wrap the patient’s mouth tightly and blow air	147	62.6
Pinch the patient’s nostril	159	67.7
Preferred training formats (multi-select)		
Regular offline practical classes	169	71.9
Expert lectures + assessment and certification	166	70.6
Online courses + simulation exercises	162	68.9
Community self-service training equipment	130	55.3
First aid knowledge competition	115	48.9

## Discussion

In this cross-sectional analysis of 235 township household health education instructors in Xinjiang, China, we found generally favorable CPR readiness and knowledge in the context of high training penetration, alongside clear, targeted gaps in procedural knowledge central to high-quality basic life support. These findings highlight a global challenge: bridging the “know-do gap” between international resuscitation guidelines and their implementation among frontline community health workers in resource-limited, culturally diverse settings.

Our finding that 87.7% of township household health education instructors had received any CPR training represents substantially higher coverage than previously reported for the general public in Chinese urban areas (6%–25.6%) (38). However, 12.4% of the trained participants relied on self-study via videos rather than hands-on practice with feedback, which lacks the deliberate practice necessary for procedural skill consolidation—a limitation observed across diverse CHW programs globally. Skill decay in CPR is well-documented internationally: psychomotor competencies deteriorate within 3–6 months without reinforcement, regardless of geographic or cultural context (39–41). Our finding that readiness decreased from 87.2 points among the participants trained ≤6 months to 48.4 points among the untrained participants aligns with this international evidence and underscores the need for spaced, high-frequency refresher training.

The workforce’s stated preferences for regular practical sessions (71.9%), expert-led certification (70.6%), and simulation-based VR (68.9%) align with international evidence on effective CHW training. Systematic reviews demonstrate that mastery learning approaches—where learners practice skills to a predefined competency threshold with deliberate feedback—yield superior retention compared to time-based training, regardless of setting (42). Low-dose, high-frequency training models, successfully piloted with community health agents in Ethiopia and Rwanda, maintain skill proficiency while minimizing workforce disruption (43–46).

Lower readiness among the Kyrgyz participants versus the Uyghur participants highlights the role of cultural and linguistic factors. Although all participants are proficient in Chinese, technical terminology and rapid instructions during demonstrations may impose additional cognitive load for those whose first language is Uyghur or Kyrgyz, leading to misunderstandings of critical parameters such as compression depth or rate ranges. Cultural norms around gender and physical contact may also affect training engagement and willingness to act: female instructors (97.4% of our sample) may face constraints when considering CPR delivery to male patients in conservative communities.

Systematic reviews and population studies have long shown the survival benefits of early bystander CPR and AED use (47–49), and guidelines emphasize compressions-first approaches, early defibrillation, and community training that is tailored to local contexts (50, 51). Educational science now favors mastery learning, debriefing, and spaced practice with feedback, strategies that are feasible at township or community health service centers (51, 52).

This study has several limitations. First, the cross-sectional design precludes causal inference. Second, self-reported knowledge may be subject to recall and social desirability bias; objective skill assessment using standardized manikin scenarios would provide more rigorous data. Third, the study population was drawn from a single region and had a highly skewed gender distribution. The extreme gender imbalance (97.4% women) reflects the occupational composition of township household health education instructors in this region, where women are preferentially recruited for roles involving household visits due to cultural norms around gender and home access. Fourth, we did not measure actual emergency response performance or patient outcomes. Fifth, the survey was administered in Chinese only, which may have disadvantaged participants whose primary language is Uyghur or Kyrgyz, despite their required Chinese proficiency. Finally, residual confounding from unmeasured variables cannot be ruled out.

## Conclusion

Among township household health education instructors in Xinjiang, China, CPR readiness and knowledge were generally favorable, although clear gaps remained in ventilation sequencing, compression depth, and AED timing. To translate these findings into improved emergency preparedness, we recommend the following: (1) mandatory quarterly low-dose, high-frequency refresher sessions with feedback-enabled manikins; (2) development and dissemination of bilingual (Uyghur, Kyrgyz, Chinese) training modules with locally relevant scenarios and cultural adaptations; and (3) expansion of AED access, coupled with integration of quarterly AED drills into routine practice. It is also recommended to equip each township health center with AEDs co-located with training manikins and advocate for public AED placement in high-traffic community venues with multilingual signage. These evidence-based strategies can strengthen the capacity of rural grassroots healthcare workers to serve as effective first responders during cardiac emergencies, ultimately reducing rural–urban disparities in OHCA survival.

## Data Availability

The original contributions presented in the study are included in the article/Supplementary material, further inquiries can be directed to the corresponding author.
