# Supplementary material for: CPR knowledge among rural grassroots healthcare workers in Xinjiang, China: a cross-sectional analysis
Source: Front Med (Lausanne). 2025 Nov 21;12:1697798. doi: 10.3389/fmed.2025.1697798 (PMC12678107; doi:10.3389/fmed.2025.1697798)
Supplement: Supplementary file 1 [file Data_Sheet_1.pdf]

Supplementary Table 1: Summary of Statistical Methods and Applications.

**Analysis Objective**	**Statistical Method**	**Application**
Compare categorical variables between groups	Chi-square test	Comparing training status, gender, ethnicity across categorical outcomes
Compare continuous outcomes between two groups	Mann-Whitney U test	Pairwise comparisons of readiness/accuracy scores
Compare continuous outcomes across ≥3 groups	Kruskal-Wallis test	Overall group differences by ethnicity, education, training recency
Identify independent correlates of readiness	Ordinary least squares (OLS) regression with HC3 robust SE	Multivariable analysis adjusting for confounders
